# Prevalence of Hepatitis B, C, and D in Germany: Results From a Scoping Review

**DOI:** 10.3389/fpubh.2020.00424

**Published:** 2020-08-28

**Authors:** Ida Sperle, Gyde Steffen, Siv Aina Leendertz, Navina Sarma, Sandra Beermann, Roma Thamm, Yanita Simeonova, Markus Cornberg, Heiner Wedemeyer, Viviane Bremer, Ruth Zimmermann, Sandra Dudareva

**Affiliations:** ^1^Department of Infectious Disease Epidemiology, Robert Koch Institute, Berlin, Germany; ^2^Charité—Universitätsmedizin, Berlin, Germany; ^3^Department of Infectious Disease Epidemiology, Translational Infrastructure Epidemiology of the German Centre for Infection Research, Robert Koch Institute, Berlin, Germany; ^4^Department of Epidemiology and Health Monitoring, Robert Koch Institute, Berlin, Germany; ^5^Centre for International Health Protection, Robert Koch Institute, Berlin, Germany; ^6^Department of Gastroenterology, Hepatology and Endocrinology, Medizinische Hochschule Hannover, Hanover, Germany; ^7^Thematic Translational Unit Hepatitis of the German Centre for Infection Research, Hanover, Germany

**Keywords:** hepatitis B, hepatitis C, hepatitis D, scoping review, epidemiology, prevalence

## Abstract

**Background:** One of the five strategic directions in the World Health Organization global health sector strategy on viral hepatitis 2016–2021 is to generate strong strategic information for focused action to understand the viral hepatitis epidemic and focus the response. Knowledge of national prevalence is a cornerstone of strategic information. Germany is considered to be a low prevalence country for viral hepatitis B, C, and D, however the prevalence is likely to be higher among at-risk groups.

**Methods:** The aim of this work was to give a detailed overview of the prevalence of viral hepatitis B (HBsAg, anti-HBc), C (anti-HCV, HCV RNA), and D (anti-HDV, HDV RNA) in different population groups in Germany. Therefore, we analyzed the results of a comprehensive literature search on various aspects of the epidemiological situation of hepatitis B, C, and D in Germany. Eligible publications including information on hepatitis B, C, and D prevalence were extracted from the overall spreadsheet table and summarized and analyzed based on virus and different population groups. A quality appraisal was performed using a checklist developed by Hoy et al. to assess risk of bias in prevalence studies.

**Results:** Overall, 51 publications were identified through the literature search. The overall prevalence of HBsAg in the general (and proxy) population ranged from 0.3 to 1.6%. Among at-risk groups, including clinical populations and health care workers, the HBsAg prevalence ranged from 0.2% (among rheumatic patients) to 4.5% among HIV positive patients. The overall prevalence of anti-HCV in the general (and proxy) population ranged from 0.2 to 1.9%. Among at-risk groups, including clinical populations and health care workers, the anti-HCV prevalence ranged from 0.04% (among health care workers) to 68.0% among people who inject drugs.

**Conclusions:** The hepatitis B and C prevalence in the general population in Germany is low. Prevalence is high to very high among at-risk populations, however for some groups evidence was incomplete or missing completely. To reach the elimination goals in Germany and implement a targeted response, more research among at-risk groups is needed.

## Introduction

Viral hepatitis (VH) is a major global public health concern. Worldwide, an estimated number of 257 and 71 million people are chronically infected with hepatitis B virus (HBV) and hepatitis C virus (HCV), respectively (1). In total, 15–20 million people are infected with hepatitis D, which corresponds to 5% among those with hepatitis B (1). In the World Health Organization (WHO) European Region, an estimated 15 and 14 million people suffer from chronic HBV and HCV infection, respectively (2).

The WHO global health sector strategy for VH (2016–2021) (3), the WHO European level action plan (2016) (2) and the German integrated national strategy for HIV, HBV, and HCV and other sexually transmitted diseases (2016) (4) represent steps forward in terms of elimination of VH. Nevertheless, they shed light on the lack of comprehensive data to monitor progress and to identify where intensified efforts are needed.

The VH viruses, HBV, HCV, and HDV, show diversity in their prevalence, but also in their modes of transmission depending on country, context, and population group. Data on the country specific epidemic in Germany as well as on population groups most at risk and the effectiveness of prevention and treatment measures are urgently needed to intensify efforts and to reach the elimination goals by 2030.

The most recent national population-based survey among adults in Germany (2008–2011) (DEGS1) found a low HBV and HCV prevalence (0.3%) (5). However, it is known that the prevalence of VH is higher in some groups more vulnerable to VH infection. More research among population groups that are often poorly represented in population-based surveys and more vulnerable to VH (hereafter populations at-risk) is needed.

The aim was to create an overview of existing evidence on the epidemiology of HBV, HCV, and HDV in different population groups in Germany in the time period from 2005 to 2017 to serve as baseline information and guide to improve monitoring of VH in Germany. In this paper, the prevalence in Germany is described.

## Materials and Methods

### Review Process

The aim of the overall scoping review was operationalised into 13 specific research questions to identify available evidence in the form of published literature on VH epidemiology in Germany (6). One of the 13 questions was “What is the prevalence of HBV, HCV, and HDV in Germany?”

The detailed methods of the review are described elsewhere (6). In brief, the search and reporting methods were based on the *reporting items for systematic reviews and meta-analysis extension for scoping reviews* (PRISMA-ScR) and the Cochrane Collaboration (https://training.cochrane.org/handbook). Included in the review were available full-text (peer- and non-peer-reviewed) publications of original works in German or English language, published between 01/01/2005 and 09/03/2017 with end of data collection after 01/01/2005 and content relevant to one or more of the research questions. The literature search was conducted in six electronic databases (MEDLINE, EMBASE, Europe PMC, Scopus, Bielefeld Academic Search Engine (BASE), and CC Med) with a detailed search string developed from the research questions Supplementary S1. The final search was conducted on 09/03/2017. The reference list of all publications retrieved from the electronic search and eligible for full-text screening as well as national surveillance reports not cited in the six electronic databases were also screened for references of further publications meeting the inclusion criteria Supplementary S2.

The screening was performed on abstract and full-text level. After full-text screening, relevant information according to the research questions was extracted from the eligible publications using standardized extraction sheets. The screening and data extraction process was performed by two independent reviewers. All discrepancies between the reviewers were discussed. A validation of the screening and extraction process was conducted.

### Data Analysis

The extracted data was assigned to different pre-defined categories based on the research questions and sorted by population groups using the definition of the target population in the corresponding publication. Population groups were defined based on the WHO guidelines on Hepatitis B and C testing (7) and adapted to the German context. Population groups were (a) the general population (GP), (b) sub-populations being representative of the national population, which are not considered at higher risk for VH and therefore act as a proxy for the GP (blood donors and pregnant women), (c) clinical populations [populations with non-VH related underlying disease and people with VH in hepatological care (PLWVH)], (d) populations at risk for VH due to risk behavior/exposure (household contacts of PLWVH, health-care workers (HCW), people living with HIV (PLWH), men who have sex with men (MSM), people in prison and closed settings, people who inject drugs (PWID), sex workers) or because they are part of a population with high VH seroprevalence (e.g., mobile or migrant populations from intermediate- and high-endemic countries). When no definition of the target population was available in the corresponding publication, the review team allocated the publication to a population group. In this paper, the evidence identified on HBV, HCV, and HDV prevalence is presented which includes all publications from the scoping review allocated to the category “prevalence.”

A quality of the evidence on prevalence was assessed using a checklist developed by Hoy et al. (8). This tool allows a judgement of the overall risk of bias based on the assessment of 10 individual items covering internal and external validity and reliability (8). The assessment was performed by one of the reviewers, and then checked by the other reviewer and categorized as either at “low risk” or “high risk” of bias. Discrepancies were discussed to reach agreement, and a third reviewer was consulted if needed. The publications were not weighted according to their quality of evidence in the analyses.

## Results

**Figure 1:**
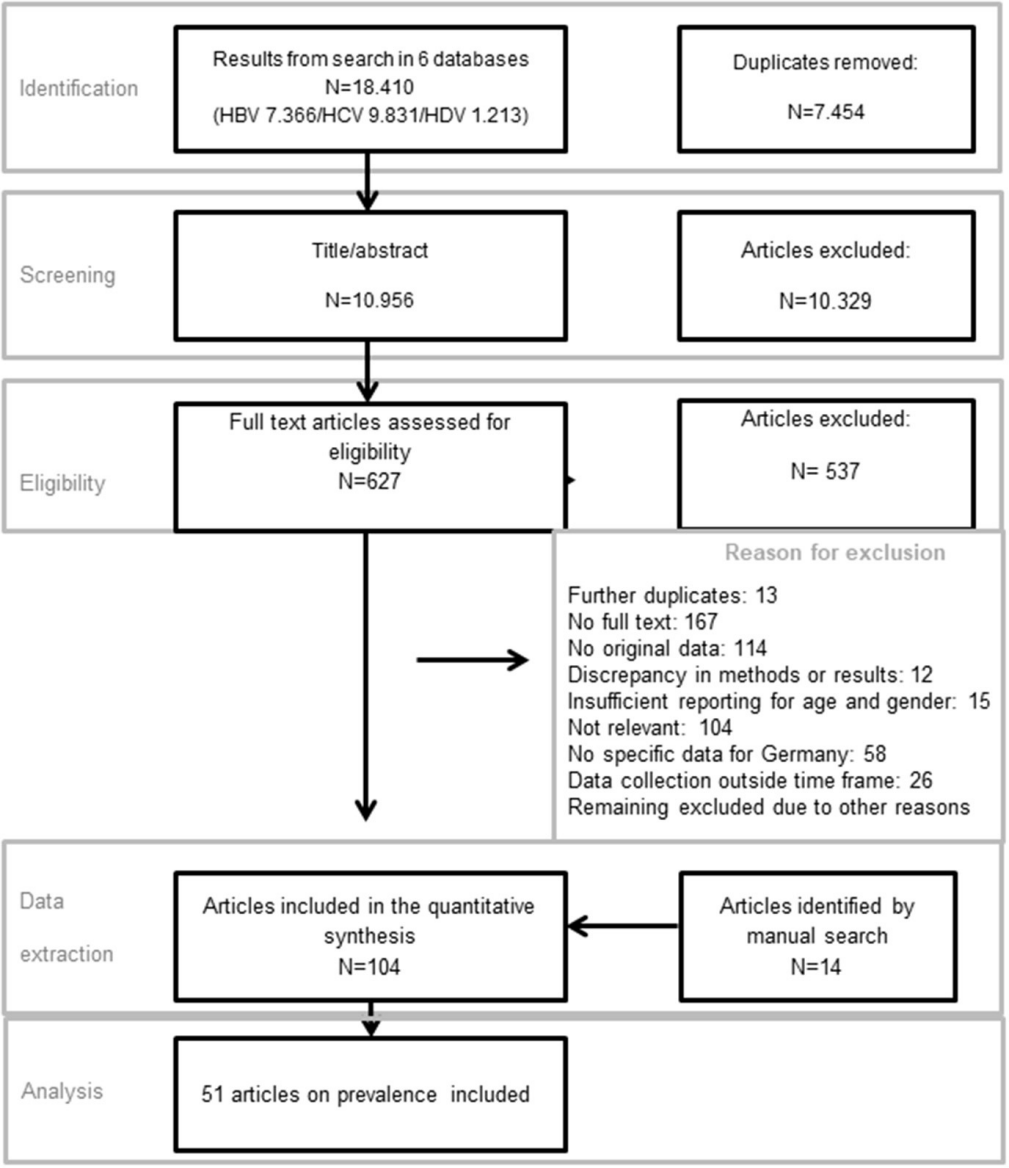
Study flow of study selection.

Overall, the electronic literature search retrieved 18,410 publications, and an additional 14 publications were identified by manual search. After removal of duplicates, abstract and full-text screening 104 publications were included in the scoping review which covered all 13 research questions. Fifty-six publications of the 104 were allocated to the category “prevalence.” Five of 56 publications were excluded due to the lack of relevance for the analysis, and the remaining 51 were included (Figure 1). Some of the included publications reported on the prevalence of more than one pathogen or marker (Table 1).

**Table 1:** Number of publications.

**Total**	**HBV**	**HCV**	**HDV**
51	39 (HBsAg: 23, anti-HBc: 19, marker not specified: 8)	33 (anti-HCV: 26, HCV RNA: 13, marker not specified: 6)	4 (anti-HDV: 1, HDV RNA: 1, marker not specified: 3)

The results of the quality appraisal performed for the publications included in this paper are summarized in Table 2. Fourteen of the 51 publications were assessed to have a high risk of bias due to either lack of properly describing their sampling and recruitment strategy, and/or that the VH markers were either not specified or that VH status was self-reported.

**Table 2:** Hepatitis B, C, and D prevalence in Germany.

**Publication (references nr.)**	**Data collection period**	**Place of data collection**	**Study type**	**Recruitment setting**	**Study population (*n*)**	**Virus**	**Mean/median age (range)**	**Prevalence**								**Risk of bias**
								**HBc-Ag**	**HBs-Ag**	**HBV DNA**	**Anti-HCV**	**HCV RNA**	**Anti-HDV**	**HDV RNA**	**Not specified**	
Knorr et al. 2015 (9)	Jan 1996–Dec 2005	Heidelberg	Cross-sectional	Hospital	Pregnant/reproducing (*N* = 5,518) (GP proxy)	HBV	(16–45 yrs)	x	1.6%	x	x	x	x	x	x	Low risk
Marcellin et al. 2015 (10)	Jan 2000–Dec 2006	Nationwide	Cross-sectional	Hospital	PLWVH in hepatologic care (chronic HCV) (*N* = 995)	HBV	48.9 yrs. (mean)	x	x	x	x	x	x	x	4.5%	High risk
Lobstein et al. 2011 (11)	2001–2006	Leipzig	Cross-sectional	Hospital	Pregnant/reproducing (*N* = 8,193) (GP proxy)	HBV	Not reported	x	0.5%	x	x	x	x	x	x	Low risk
Alba-Alejandre et al. 2009 (12)	2001–2008	Munich	Cross-sectional	Clinic (all women who gave birth in clinic; HBsAg collected retrospectively) (medical records, serology)	Pregnant/reproducing women (*N* = 15,873) (GP proxy)	HBV	Not reported	x	0.8%	x	x	x	x	x	x	Low risk
Cai et al. 2011 (13)	May 2003–2006	Nationwide	Cross-sectional	At physicians and *via* self-completed questionnaires	GP (children) (*N* = 13,065)	HBV	Not reported (3–17 yrs.)	0.5% [CI: 0.4–0.7]	38.7% [95% CI 20.0–57.5] (of the 0.5%)	x	x	x	x	x	x	Low risk
Hüppe et al. 2008 (14)	Mar 2003–May 2006	Nationwide	Cohort	Hepatitis centers and outpatients units	PLWVH in hepatologic care (chronic HCV) (*N* = 10,326)	HBV	43.4 yrs. (mean)	x	x	x	x	x	x	x	1.5%	High risk
Ernst et al. 2012 (15)	Aug 2004–2008	Potsdam	Cross-sectional	Hospital	Hospital patients but not only hepatitis related patients(Clinical population) (*N* = 803)	HBV	61 yrs. (mean)	x	1.9%	x	x	x	x	x	x	Low risk
Zeiler et al. 2006 (16)	2005	Not reported	Surveillance	German blood donation services	Blood donors (GP proxy) (*N* = 3964)	HBV	Not reported	0.9% [95% CI 0.8–1.4]	0%		x	x	x	x	x	Low risk
Deterding et al. 2012 (17)	Not specified (a collaboration project of Northern Expert Network for Hepatitis established 2005–2007)	Hannover	Cross-sectional	Hospital/treatment centers	Child/partner of chronic HBV patients (*N* = 312) (at-risk population)	HBV	42 yrs. (mean)	x	x	x	x	x	x	x	10.7%	High risk
Walch 2010 (18)	2/2006–11/2007	Baden-Württemberg/Hesse	Cross- sectional	5 Transfusion centers of the blood donation service in Baden-Württemberg/Hesse provided blood samples of blood donors	Blood donors (GP proxy)	HBV	Not reported	1.4%	x	0.1%	x	x	x	x	x	Low risk
Wiese et al. 2014 (19)	2011–2012	East Germany (Leipzig, Dresden, Rostock, Chemnitz, Potsdam, Berlin, Magdeburg, Cottbus, Jena, Erfurt, Halle)	Cohort	Referral centers, multi-centric	PLWVH in care (HCV) (*N* = 718)	HBV	At HCV-infection: 24 yrs. (median), after 35 yrs.: 57 yrs. (median)	x	x	x	x	x	x	x	0.1%	Low risk
Claus et al. 2016 (20)	Aug 2010–2012	Rhineland-Palatinate	Cross-sectional	Schools for handicapped (*n* = 13) (questionnaires)	Health care staff (staff at the schools) (*N* = 367)	HBV	45 yrs. (mean) (not reported)	1.7%	51.8%	x	x	x	x	x	x	Low risk
Feuchtenberger et al. 2016 (21)	2011–2015	Würzburg	Cross-sectional	Hospital, single center	Clinical population (rheumatic disease) (*N* = 1,338)	HBV	60.98 yrs. (mean)	5.6%	0.2%	x	x	x	x	x	x	High risk
Kartashev et al. 2016 (22)	2011–2015	Cologne	Cross-sectional	University hospital	PLWVH in hepatologic care (chronic HCV) (*N* = 1208)	HBV	Not reported	x	x	x	x	x	x	x	39.1%	High risk
Mockenhaupt et al. 2016 (23)	Oct 2013–Nov 2015	Berlin	Cross-sectional	Clinic (*n* = 1)	Migrants (unaccompanied minors) (*N* = 488) (at-risk population)	HBV	(6–17 yrs.)	x	x	x	x	x	x	x	0%	High risk
Hampel et al. 2016 (24)	Aug 2015	Northern Germany	Cross-sectional	Central refugee stations (*n* = 6)	Migrants (refugees) (*N* = 793) (at-risk population)	HBV	28.8 yrs. (median) (3–76 yrs.)	14.0% (95% CI:11,9–16,9)	2.3% (95% CI: 1,3–3,4)	x	x	x	x	x	x	Low risk
Jansen et al. 2015 (25)	Jun 1996–May 2012	Nationwide	Cohort	Clinics	MSM (HIV positive) (*N* = 1,838) (at-risk population)	HBV, HCV	33 yrs. (mean age at HIV seroconversion) (17–76 yrs.)	28.8%	x	x	8.2%	4.0%	x	x	x	Low risk
Winkelmann et al. 2016 (26)	Jan 1997–Dec 2008	Hannover	Cross-sectional	Hospital, Hannover Medical School, trauma department (*n* = 1)	Clinical population (*N* = 1,373)	HBV, HCV	64.2 yrs. (mean)	x	0.7%	x	2.0%	x	x	x	x	Low risk
Wiegand et al. 2009 (27)	2000–2005	Nationwide	Cross-sectional	21 transfusion centers throughout Germany	Autologous blood donors (clinical population) (*N* = >35,000)	HBV, HCV	Not reported	x	0.2% [95% CI 0.1–0.2] East 0.3% [95% CI 0.2–0.4] West	x	0.2% [95% CI 0.1–0.3] East 0.3% [95% CI 0.3–0.4] West	x	x	x	x	Low risk
Reuter et al. 2011 (28)	Jan 2001–Dec 2005	Cologne and Düsseldorf	Cross-sectional	University Hospitals	HIV positives (*N* = 918) (at-risk population)	HBV, HCV	37 yrs. (median) (17–77)	42.8%	4.5%	x	10.6%	x	x	x	x	High risk
Wicker et al. 2007 (29)	Winter semester 2005/2006	Frankfurt	Cross-sectional	University hospital	Health care workers (*N* = 223)	HBV, HCV	23.4 yrs. (mean) (20–45 yrs.)	0.9%	x	x	0%	x	x	x	x	High risk
Offergeld et al. 2007 (30)	2005	Nationwide	Surveillance data	All blood donor centers provide data on demographics/test results of routine testing.	Blood donors (GP proxy) (*N* = 452,670, new donors)	HBV, HCV	Not reported	0.1%			0.1%		x	x	x	Low risk
Willand et al. 2008 (31)	2006	Nationwide	Surveillance	German Blood Donor Centers	Blood donors (GP proxy) (*N* = 512,023 first donors)	HBV, HCV	Not reported	0.2%			0.1%		x	x	x	Low risk
Offergeld et al. 2010 (32)	2007	Nationwide	Surveillance data	All blood donor centers provide data on demographics/test results of routine testing.	Blood donors (GP proxy) (*N* = 548,608 new donors)	HBV, HCV	Not reported	0.1% (2008), 0.1% (2009), 0.1% (2010)			0.1% (2008), 0.1% (2009), 0.1% (2010)					Low risk
Wicker et al. 2009 (33)	Apr–May 2007	Frankfurt	Cross-sectional	University hospital	Health care workers (*N* = 366)	HBV, HCV	24.4 yrs. (mean) (19.8–48.2 years.)	0.5%	x	x	0.3%	x	x	x	x	High risk
Müller et al. 2009 (34)	Feb 2008–Dec 2008	Munich	Cross-sectional	Specialized methadone substitution center in Germany	PWID (*N* = 146) (at-risk population)	HBV, HCV	35 yrs. (mean)	x	x	x	68.0%	28.0%	x	x	1.3% (chronic HBV)	High risk
Offergeld et al. 2012 (35)	2008–2010	Nationwide	Surveillance data	All blood donor centers provide data on demographics/test results of routine testing.	Blood donors (GP proxy) (N=570,852)	HBV, HCV	Not reported	0.1%			0.1%		x	x	x	Low risk
Poethko-Müller et al. 2013 (5)	2008-2011	Nationwide	Cohort	Population-based. Participants were the invited to fill out questionnaire and visit examination clinics (DEGS1)	GP (*N* = 7,047)	HBV, HCV	Not specified (18–79 yrs.)	0.3% [0,2–0,6], 0.6% (only Anti-HBc)	x	0.3% [95% CI 0.1-0.5]	0.2%	x	x	x	Low risk	
Baars 2011 (36)	2009–2010	Lower Saxony	Cross-sectional	Company doctors (all medical staff in company doctor practices invited to participate in survey, self-reported)	Health care workers (HBV: *N* = 831, HCV: *N* = 2295)	HBV, HCV	Not reported	1.6% (self-reported)	x	x	0.0% (self-reported)	x	x	x	x	Low risk
Baid-Agrawal et al. 2014 (37)	2009–2011	Berlin	Case-control	Outpatient transplant clinic, Charité University Hospital (medical records, serum sampling)	Kidney transplant recipients (clinical population) (*N* = 417)	HBV, HCV	53.0 yrs. (mean) (53.0 yrs. +/−12.8)	*x*	*3.4%*	*x*	*4.8%*	*4.6%*	*x*	*x*	*x*	High risk
					Chronic haemodialysis patients) (*N* = 417) (clinical population)	HBV, HCV	66.1 yrs. (mean) (66.1 yrs. +/– 14.9)	x	0.5%	x	3.6%	2.4%	x	x	x	
Schmidt et al. 2013 (38)	Sep 2009–Mar 2011	Hamburg	Cross-sectional	Hospital	Alcohol dependent (*N* = 463) (clinical population)	HBV, HCV	Not reported	8.3% [95% CI: 5.7–10.8%]	x	x	5.2% [95% CI: 3.2–7.2%]	3.2%	x	x	x	Low risk
Darstein 2015 (39)	Aug 2010–Nov 2011	Berlin	Cross-sectional	Accident and emergency unit, hospital (*n* = 1)	Emergency department patients (Clinical population) (*N* = 1,942)	HBV, HCV	59.5 yrs. (median) (18–97 yrs.)	0.5% [95% CI: 0.2–0.8] (anti-HBc & HBsAg), 9.9% [95%CI 8.6–11.3%] (anti-HBc), 6.1% [95% CI:5.0–7.2], (anti-HBc and anti-HBs), 1.9% [95% CI: 1.3–2.5] (anti-HBc and anti-HBs negative)		x	0.9% [95% CI 0.5–1.3]	0.5% (HCV RNA)	x	x	x	Low risk
Heidrich et al. 2014 (40)	Nov 2010–Jan 2012	North-Western Germany	Cross-sectional	Primary care centers (*n* = 8)	Migrants (*N* = 1,298) (at-risk population)	HBV, HCV	49.1 yrs. (mean) (49.1 +/– 15.8 yrs.)	32.5%	3.6%	2.2%	1.9%	0.7%	x	x	x	Low risk
Mone 2015 (41)	Jan 2011–Mar 2011	Aachen, Berlin, Bochum, Cologne, Essen/Hamm, Hamburg, Frankfurt am Main, Münster, Saarbrücken, Wuppertal	Cross-sectional	On the street and in drug consumption places	PWID (“street clients”) (*N* = 420) (at-risk population)	HBV, HCV	38.4 yrs. (mean) (38.4 +/– 8.4 yrs.)	x	x	x	x	x	x	x	14.1% (HBV+), 58.3% (HCV +) (self-reported)	High risk
				Substitution clinics (*n* = 12)	PWID (“substitution patients”) (*N* = 404) (at-risk population)	HBV, HCV	40.8 yrs. (mean) (40.8 +/– 8.6 yrs.)	x	x	x	x	x	x	x	14.0% (HBV +), 58.7% (HCV +) (self-reported)	
Kant et al. 2016 (42)	Feb 2011–Jan 2012	Leipzig	Cross-sectional	Hospital, department of internal medicine and neurology	Baby boomers (born 1955–1965) (*N* = 1,235) (GP proxy)	HBV, HCV	(only available for GP)	x	0.6%	x	1.5%	x	x	x	x	Low risk
					GP (*N* = 6011)	HBV, HCV	62.4 yrs. (mean)	x	0.7%	x	0.9%	x	x	x	x	
Bremer et al. 2016 (43)	2011–2014	Berlin, Cologne, Essen, Frankfurt am Main, Hamburg, Hannover, Leipzig, Münich	Cross-sectional	Low threshold drug services^a^ (questionnaires, serology)	PWID (*N* = 2,077) (at-risk population)	HBV, HCV	38.0 yrs. (median) (17–65 yrs.)	25.0%	0.1%	x	66.0%	44.0%	x	x	x	Low risk
Wolffram et al. 2015 (44)	Jan 2012–Jun 2013	North Rhine Westphalia	Cross-sectional	General practitioner practices (*n* = 51)	GP (*N* = 21,008)	HBV, HCV	57.5 yrs. (mean) (7–107 yrs.)	x	0.5%	x	1.0.%	0.4%	x	x	x	Low risk
Wicker et al. 2016 (45)	Feb 2014–Jan 2015	Frankfurt/Main	Cross-sectional	Accident and emergency unit, University Clinic Frankfurt	Clinical population (*N* = 275)	HBV, HCV	46.7 yrs. (mean) (8–91 yrs.)	x	0.7%	x	2.6%	x	x	x	x	Low risk
Bert et al. 2016 (46)	2016	Frankfurt am Main	Cohort	Emergency department of hospital (medical records)	Emergency department patients (Clinical population) (*N* = 10,215)	HBV, HCV	59.0 yrs. (mean) (24–94 yrs.)	x	1.3%	x	2.7%	x	x	x	x	High risk
Schmidt et al. 2011 (47)	2006	Nationwide	Cross-sectional	Online survey	MSM (*N* = 4,385) (at-risk population)	HCV	32 yrs. (median, HIV-neg/not tested) and 40 yrs. (median, HIV-pos.) (16–79 yrs.)	*x*	*x*	*x*	2.4% (HIV negative/untested: 0.8%), HIV positive: 8.8%)	*x*	*x*	*x*	*x*	Low risk
Schulte et al. 2009 (48)	Mar 2006 (not further specified)	Nationwide	Cross-sectional	Prison	People in prisons (*N* = 14,187) (of which 21.9% (*n* = 3,111) were PWID) (at-risk population)	HCV	Not reported	x	x	x	x	x	x	x	*14.3%*	High risk
Tomeczkowski et al. 2015 (49)	2007–2011	Nationwide	Cohort	Health insurance	GP proxy (*N* = 5 464,191)	HCV	Not reported	x	x	x	x	x	Projected prev.: Average of 0.2% per year and 0.2% over three years; 19.0% of the patients were first diagnosed with acute hepatitis.	x	Low risk	
Thönnes et al. 2017 (50)	2007–2013	Nationwide	Cohort	German company health insurance funds	GP proxy (*N* = 3,200,000 million)	HCV	Not reported	x	x	x	x	x	x	x	0.2% (projected prevalence)	Low risk
Vermehren et al. 2012 (51)	May 2008–Mar 2010	Berlin Frankfurt/Main	Cross-sectional	Hospital emergency units	Clinical population (*N* = 28809)	HCV	51.9 yrs. (mean) (31.9–71.9)	x	x	x	2.6% [95% CI 2.4–2.8]	1.6% [95% CI 1.5–1.8]	x	x	x	Low risk
Dogiami 2014 (52)	Jun 2009–Jun 2010	Bochum	Cross-sectional	Hospital, accident and emergency unit of the St. Josef Hospital	Clinical population (*N* = 8,435)	HCV	51.15 yrs. (median) (10–100)	x	x	x	3.5%	1.6%	x	x	x	Low risk
DiBonaventura et al. 2012 (53)	2010	Nationwide	Cross-sectional	Online survey (Self-reported)	GP (*N* = 15,070)	HCV	Not reported (18–65 yrs.)	x	x	x	x	x	x	x	0.4% (self-reported (projected)	High risk
Jablonka et al. 2017 (54)	Aug 2015	Northern Germany	Cross-sectional	Reception center for refugees (*n* = 6)	Migrants (*N* = 236) (at-risk population)	HCV	28.7 yrs. (mean) (3–76)	x	x	x	0.4%	x	x	x	x	Low risk
Magistro 2014 (55)	Jan 2000–Dec 2008	Ulm	Cross-sectional	Hepatology Unit, University Hospital	PLWVH in hepatologic care (chronic HBV) (*N* = 327)	HCV, HDV	41.9 yrs. (mean) (17–81 yrs.)	x	x	67.7% 19.6% *(HBeAg)*	x	x	x	x	0% (HCV, HDV)	Low risk
Erhardt 2010 (56)	Jan 1989–Dec 2008	Düsseldorf	Cohort	Hospital	PLWVH in hepatologic care (chronic HBV) (*N* = 1,307)	HDV	Not reported	x	x	x	x	x	5.1% (chronic HDV)	x	x	Low risk
Reinheimer et al. 2012 (57)	Jan 2000–Oct 2011	Frankfurt am Main	Cross-sectional	University Hospital Frankfurt	PLWVH in hepatologic care (chronic HBV) (*N* = 2,844)	HDV	Not described	x	x	x	x	x	7.4%	64.5%	x	Low risk
Fischer et al. 2012 (58)	Dec 2004–Mar 2007	Nationwide	Cross-sectional	German centers with a predominantly hepatologic focus (*n* = 74)	PLWVH in hepatologic care (chronic HBV) (*N* = 1,535)	HDV	39.8 yrs. (mean) (38.9 +/– 13.6 yrs.)	*x*	*x*	*x*	*x*	*x*	*x*	*x*	*1.4%*	Low risk

a *Harm reduction-based health care service with minimal demands for their clients*.

### Prevalence of Hepatitis B, C, and D in Germany

Of the 51 publications reporting on VH prevalence 37 had a cross-sectional design, eight a cohort design, five were surveillance studies, and one was a case-control study (37). For seven publications the origin of the data was national surveillance. National level data were reported by 16 publications, while regional or local level data were reported by the remaining publications, except one which did not report on which level the data were from (16).

#### Hepatitis B

The 39 publications covering HBV prevalence were on studies conducted between 1996 and 2016. The prevalence of HBV in the GP, including proxy populations, was reported in 13 publications of which 11 were at national level (5, 10, 13, 25, 27, 30–32, 35, 58, 59). One publication did not describe on which level the data were from (16).

The prevalence of HBsAg in the GP ranged from 0.3 to 0.7%, and 0 to 1.6% among proxy populations. The prevalence of anti-HBc ranged from 0.5 to 0.6% in GP, and 0.9 to 1.4% in proxy populations. Six publications (16, 18, 30–32, 35) included surveillance data among blood donors. Four of these reported on the prevalence of HBsAg, HBcAg, HBV-DNA (not separately) among first time blood donors and reported a range from 0.12 to 0.15%. Two studies described anti-HBc prevalence among first time blood donors and found a prevalence of 1.9% (18) and 0.9% (16).

Four studies described anti-HBc prevalence among HCWs which ranged from 0.5 to 1.7%, one identifying a self-reported anti-HBc prevalence among medical doctors (1.6%) (36).

One study included HBV infection among household contacts (partner and children) and reported a self-reported prevalence of 10.7% (17).

**Figure 2:**
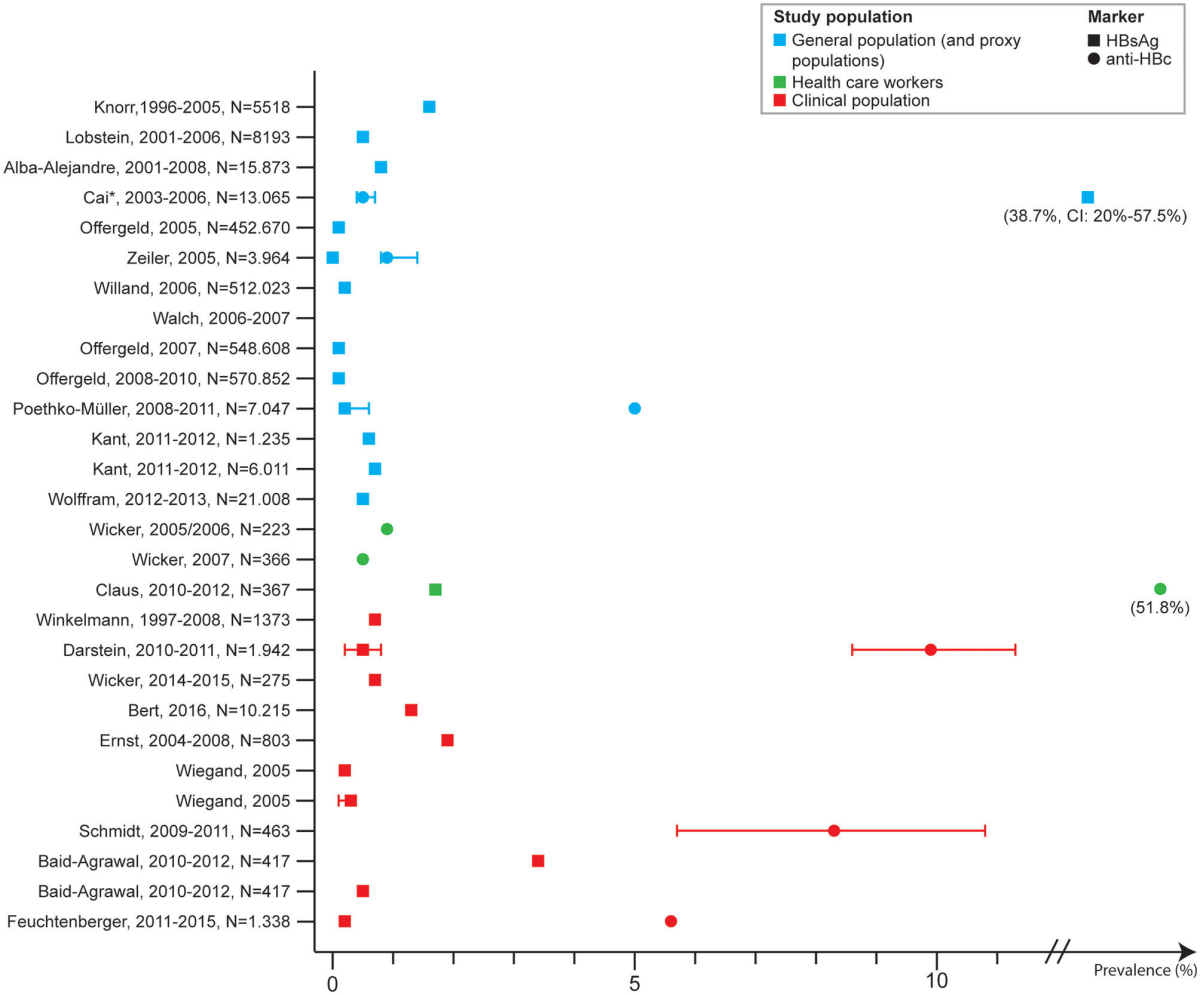
Hepatitis B prevalence in Germany by study population and marker, 2005–2017.

Thirteen studies looked at HBV prevalence among clinical populations, of which four were VH patients in hepatologic care. These four described the proportion of patients with HCV who were co-infected with HBV (markers not specified) which ranged from 0.1 to 39.1%. The prevalence of HBsAg was reported by eight studies and ranged from 0.2 to 3.4%. Four of these were among emergency and trauma department in which the prevalence ranged from 0.5 (anti-HBc and HBsAg) to 1.3%. One study reported an anti-HBc IgG prevalence of 8.3% among alcohol dependent patients (38), and one study an anti-HBc prevalence of 5.6% among patients with rheumatic disease (21) (Figure 2).

Eight publications described HBV prevalence in at-risk populations, and three were among people with migration background. One study was among refugees screened in an emergency department and found a prevalence of HBsAg and anti-HBc of 2.3 and 14.0%, respectively. The country of birth was not specified (24). Another study screened patients with migration background (patient or parents of patient) and found an HBsAg prevalence of 3.6% and anti-HBc of 32.5%. In total, 87.3% of the patients were from the Eastern Mediterranean Area, 12.0% were from Eastern Europe, and 0.7% originated from other countries (40). The third study tested 488 Syrian refugees upon arrival in Germany, but none were HBV positive (markers not specified) (23).

HBV prevalence among PLWH was reported by two studies, one of which was among HIV positive MSM. The prevalence of HBsAg was 4.5% among HIV patients (28) and 1.7% among HIV positive MSM (25).

**Figure 3:**
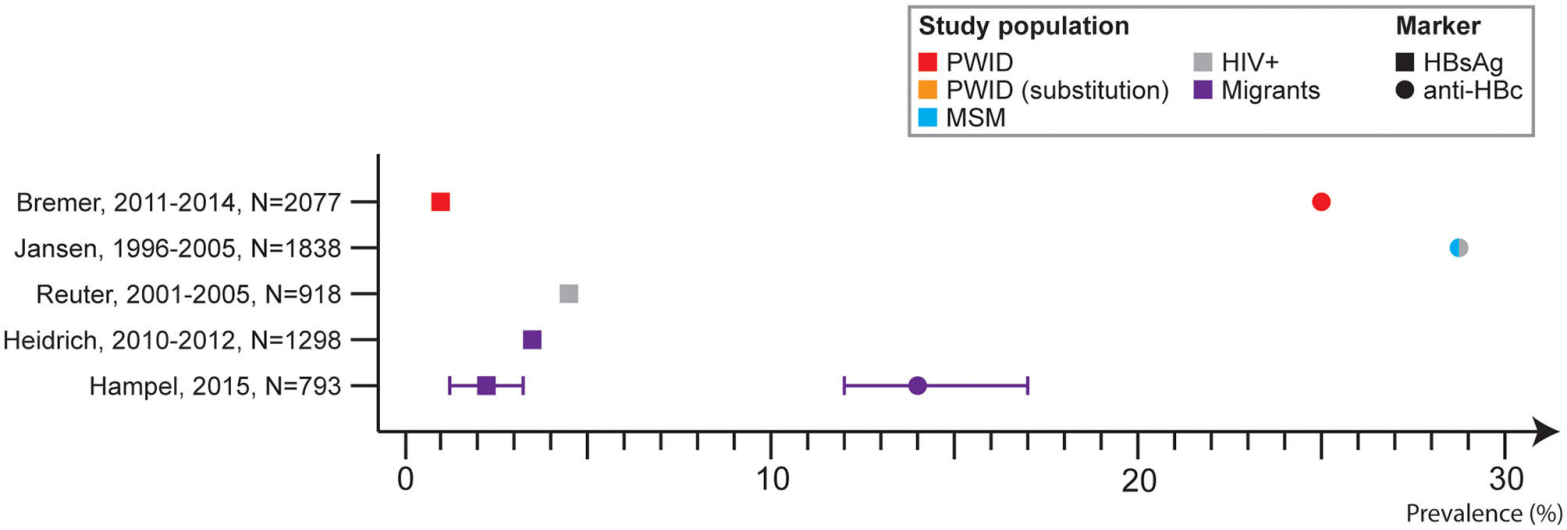
Hepatitis B prevalence in Germany by study population (at-risk) and marker, 2005–2017.

Three studies included results on HBV prevalence among PWID. One study included results on self-reported HBV infection among PWID recruited from the streets or drug consumption rooms and from substitution clinics, and found a rate of 14.1 and 14.0%, respectively (41). One study reported an HBsAg prevalence of 1.3% among PWID in specialized methadone substitution centers (34) and another reported an anti-HBc prevalence of 25% (range in the cities: 4.6–33%), among which 1.1% were HBsAg positive (range in the cities: 0.3–2.5%). among PWID recruited from low threshold services (43) (Figure 3).

#### Hepatitis C

The 33 publications covering HCV prevalence were on studies conducted between 1996 and 2014. The prevalence of HCV in the GP, including proxy populations, was reported in 11 publications of which 10 were on the national level.

The anti-HCV prevalence in the GP ranged from 0.2 to 1.9%, and was 1.5% among baby boomers (proxy population) (42). Two studies reported an HCV RNA prevalence of 0.2% (5) and 0.4%, respectively (44).

Four publications (30–32, 35) reported on surveillance data among blood donors, describing an anti-HCV range from 0.06 to 0.08%.

Three studies on prevalence of anti-HCV among HCWs reported a prevalence of 0 and 0.03% (29, 33) and of 0.04% self-reported anti-HCV (36).

**Figure 4:**
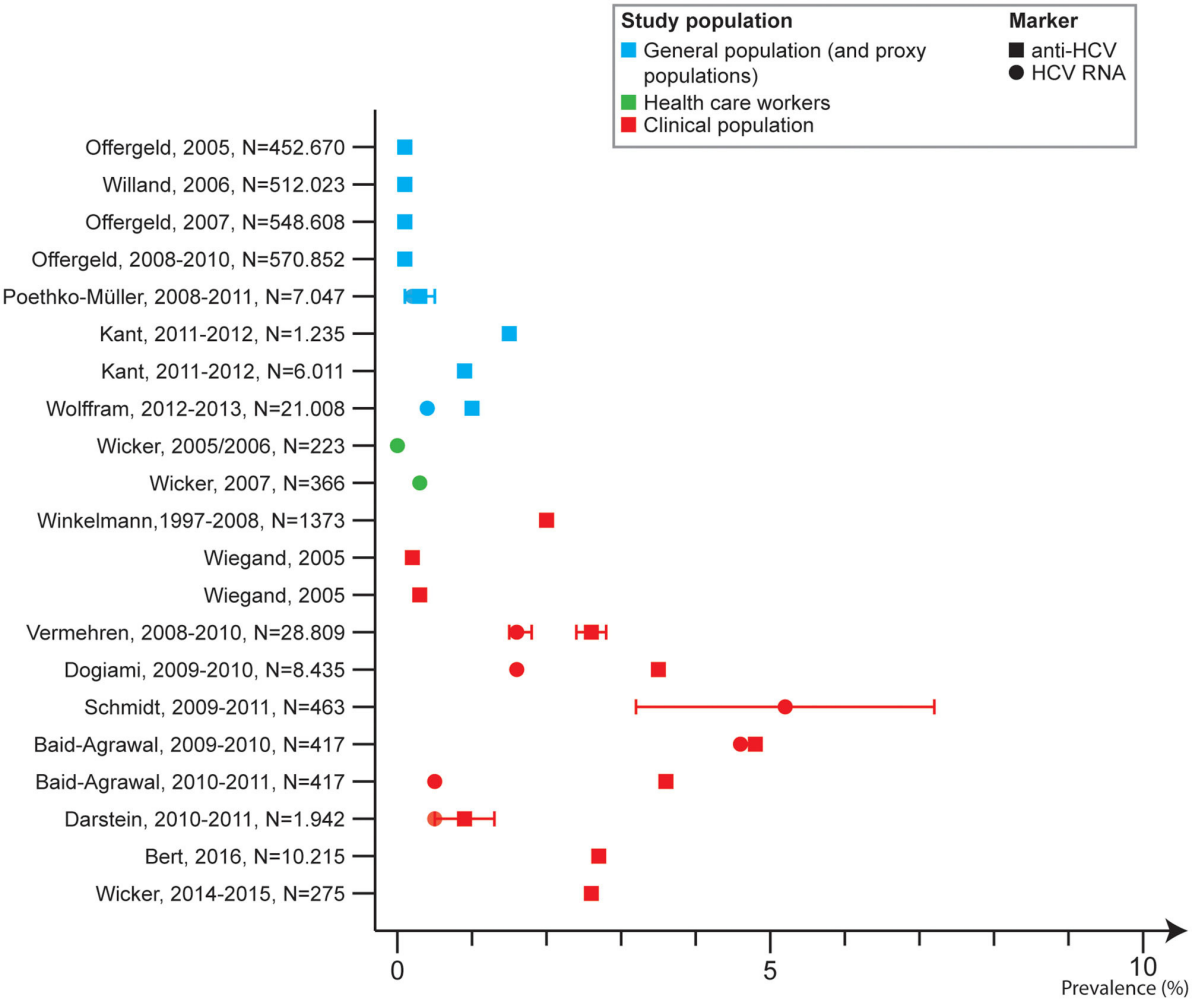
Hepatitis C prevalence in Germany by study population and marker, 2005–2017.

Ten studies analyzed HCV prevalence among clinical populations (26, 27, 37–39, 45, 46, 51, 52, 55), including one in HBV patients in care (55). Anti-HCV ranged from 0.2 to 5.2% with the lowest prevalence in autologous blood donors (giving blood for themselves). One study reported on HCV RNA among two groups of clinical patients and reported a prevalence of 2.4% among chronic haemodialysis patients and 4.6% among kidney transplant recipients (37). One study reported on the proportion of HBV patients with HCV without specifying the marker where 0% were co-infected with HCV (55). Six studies were among emergency and trauma department in which the anti-HCV prevalence ranged from 0.9 to 3.5% (Figure 4).

Nine publications reported HCV prevalence in at-risk populations. Two studies (40, 54) reported an anti-HCV prevalence among mobile/migrant populations of 1.9% among patients with migration background in eight primary care centers in Northwest Germany (40) and 0.4% among refugees and asylum seekers who went through routine screening for infectious diseases upon arrival in Germany (54). The first study with patients largely originating from the Eastern Mediterranean area (87.3%) followed by Eastern Europe (12.0%) and other countries (0.7%) also reported an HCV RNA prevalence of 0.7% (40). The country of origin of the refugees and asylum seekers in the second study was not described (54).

Three studies reported on HCV prevalence among PWID (34, 41, 43) in which the anti-HCV prevalence ranged from 63.0 to 68.0%. One cross-sectional study covered eight cities where the anti-HCV prevalence ranged from 36.9% in Leipzig to 73.0% in Hannover. The HCV RNA prevalence ranged from 23.1 to 54.0% (43).

One study included results on self-reported HCV prevalence among PWID recruited from the street and PWID in opioid-substitution treatment (OST) and found a prevalence of 58.3 and 58.7%, respectively (41). One nationwide study including 21 prisons found an HCV prevalence reported by prison physicians of 14.3% among people in prisons, of which 21.9% were PWID (48).

**Figure 5:**
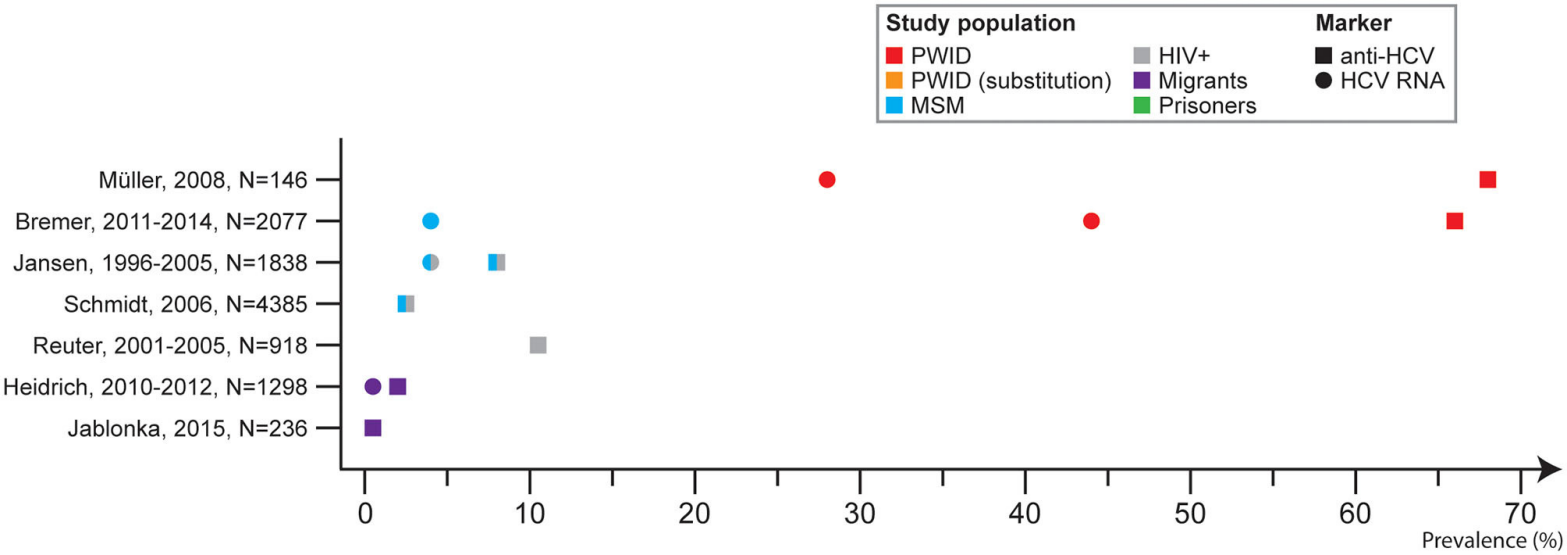
Hepatitis C prevalence in Germany by study population (at-risk) and marker, 2005–2017.

Three studies described prevalence among PLWH, and for two studies these were MSM. Among HIV positive patients the anti-HCV prevalence was 10.6% (28). Among MSM with HIV the anti-HCV prevalence was 8.2% (25). One study described self-reported HCV prevalence among MSM who were HIV positive and HIV negative (or untested) and the prevalence was 8.8 and 0.2%, respectively (47) (Figure 5).

#### Hepatitis D

Four publications covered HDV prevalence based on studies conducted between 1989 and 2011. All four included results on the prevalence in patients with chronic HBV, three recruited patients from hospital settings and in one physicians provided patient data (58). The overall prevalence of HDV ranged from 0 to 7.4%. One study specified the HDV marker and reported an anti-HDV prevalence of 7.4%, and HDV RNA of 64.5% (57). One study collected nationwide data from 74 centers across Germany with focus on hepatology and the prevalence was 1.4% in the population of HBV positives.

## Discussion

The aim of this analysis was to assess the state of evidence on HBV, HCV, and HDV prevalence in Germany. To our knowledge this is the first time that all available evidence on HBV, HCV, and HDV prevalence has been systematically searched for. The results demonstrate that there is a large body of evidence on prevalence of HBV and HCV in Germany, but less on HDV.

The available evidence is highly variable. Good coverage was found for the GP and some clinical populations but there are gaps in knowledge for some at-risk populations and missing for sex workers, people who have received blood transfusion and persons with tattoos/piercings.

### Prevalence in General Population

The low prevalence of HBV and HCV found in the GP is similar to what has been found in other European countries (60). A higher prevalence was found for proxy populations for the GP [e.g., some patient groups or among pregnant women compared to larger health examination surveys which use a random sample of the GP such as DEGS1 (5)]. The robustness of estimates based on proxy populations for the GP has its limitations. On the one hand, pregnant women may serve as a good proxy as women with migration background are likely to be better represented, however in some cases even over-represented, compared to the larger population-based surveys. On the other hand, they represent a group in more frequent contact with health care services and women of younger age only, and not all pregnant women attend all routine screenings potentially introducing selection bias.

A higher prevalence can be found in birth cohorts of the GP exposed through nosocomial or transfusion-related transmission. These are often referred to as baby boomers. Although the epidemiology is changing with injecting drug use now being a primary risk factor, the prevalence of VH is associated with age and sex. A higher prevalence is often found among males and with increasing age (5, 61, 62), and using baby boomers as proxy population for the GP in Germany should be carefully considered. The study which compared baby boomers and GP in this study found similar prevalence of HBsAg prevalence in the two groups, but higher anti-HCV prevalence among the German baby boomer population (42). Data from first time blood donors were included as this group is more likely to resemble the GP compared to multiple blood donors.

The prevalence of HDV was above 5% in two of the four studies identified. While HDV is relatively rare compared to HBV and HCV, as it requires the envelope of HBV for its entry into hepatocytes, it has important implications for mortality and morbidity (56). The prevalence of HDV found in this review is similar to that in other European countries (e.g., Spain (4.0%) (63), and Switzerland (4.4%) (64), but lower than for example in Italy with 11.9% (65)). A recent systematic review found that globally 10.6% of HBsAg carriers without risk factors (IDU or high risk sexual behavior) are infected with HDV, but higher prevalences were found among those with risk factors with 37.6% in PWID and 17.0% in populations with high risk sexual behavior (66).

Improving screening for people with migration background from areas of high prevalence (e.g., from Turkey, who represent the majority of migrant populations in Germany and which is a high prevalence area (67)), may improve early diagnosis, treatment, and data on HDV in Germany.

### Prevalence in At-Risk Populations

The VH burden disproportionately affects some population groups more (61) which was also confirmed in this review.

Sexual transmission of HBV is more common than of HCV, whereas HCV is largely transmitted via blood-to-blood contact with infected fluids. The most common transmission paths ultimately affect which groups are at highest risk and where there is the highest prevalence (68, 69).

The HBV prevalence among populations with migration background was higher than in the GP among refugees who were screened in an emergency department (country of origin not specified) (24), and among patients with migration background primarily from the Eastern Mediterranean Area and Eastern Europe (40). The study among refugees arriving from Syria, where none were tested positive for HBV (23) was among unaccompanied minors who may have a different prevalence than the adult population.

The reasons for a higher prevalence found in the two studies are likely multifacetted. Firstly, people with migration background and refugees are two groups of people that need to be distinguished. Refugees are more likely to have been exposed to risks during flight from war and or persecution in home country and to have lack of access to well-functioning health care services and timely medical care. For people with migration background, prevalence will depend in part on the prevalence in the country of origin. This was however only described in two of three studies (23, 40). The wide ranges of prevalence (from 0 to 3.6%) found in this review coincide with results from other European countries demonstrating large heterogeneity depending on country of origin, ranging from 0 to 22.2% among mobile/migrant populations with the highest prevalence reported among migrants from countries in Southeast Asia (20%) and Sub-Saharan Africa (22.2%) (70). The highest rates of prevalence were found among refugees from east European (1.6–53.1%) and Southeast Asian countries (57.7%) (70).

For HCV, a relatively lower prevalence than HBV and closer to that of the GP was found for people with migration background. One study (not with specific focus on people with migration background) looking at HCV prevalence among patients arriving at an emergency room observed that 67.8% of those HCV positive were of German origin (51).

In one study of 236 refugees and asylum seekers screened for anti-HCV at a reception center upon arrival in Germany, one tested anti-HCV positive (54) (country of origin not specified), and in the other 1.9% were anti-HCV positive among 1,298 people with migration background, primarily from the Eastern Mediterranean Area and Eastern Europe, tested in primary care centers (40). The most HCV affected regions are the WHO Eastern Mediterranean and European Regions (71), corresponding to the higher prevalence found among the people from the Eastern Mediterranean Area and Eastern Europe. HCV estimates from other European countries range from 0 to 19.2% with the highest prevalence rates reported among migrants from countries in Eastern Europe (9.3%) and Sub-Saharan- Africa (19.2%). Among refugees, the highest rates were found among refugees from South Asia (9.1%) and Sub-Saharan Africa (26.7%) (70).

Factors relating to higher vulnerability as a result of a migration background are intertwined and related to social and political factors, either in the country of origin or the new country. Further, drawing any general conclusions for migrants based on this review is challenging. The country of origin differed in the included publications ranging from patients with parents with migration background to newly arrived refugees from Syria. Moreover, the publications that reported on prevalence among mobile/migrant populations categorized the countries/regions of origin differently. A standardization of countries/regions of origin reported in literature would improve the comparison across countries and over time to improve the understanding of the epidemic. Moreover, strengthening the terminology is crucial, as different terminology has very distinct and different meanings, and confusing these terms (e.g., migrant vs. refugee, or nationality vs. country of residence) hinders standardization of data and generation of comparable estimates.

More efforts are needed to reach migrant/mobile populations in the larger health surveys conducted in Germany and to include VH testing in these larger population-based surveys. This is currently being piloted and planned to be implemented at the Robert Koch Institute (RKI) as part of the Improving Health Monitoring in Migrant Populations (IMIRA) Project (72, 73).

People living with HIV (PLWH) are also disproportionately affected by VH, and higher rates of HBsAg prevalence was found among PLWH in this review. Sexual transmission of HBV may occur in particular among MSM and/or heterosexual persons with multiple sex partners, making the interaction between different at-risk groups important to consider.

A higher prevalence of HCV among PLWH was also found, which mirrors the global pattern where a 5.8 times (95% CI 4.5–7.5) increased odds of HCV antibody positivity in HIV-positive people compared with HIV-negative people across all population groups has been documented (74). There is particularly a high rate among at-risk groups with rates as high as 6.4% in MSM and 82.4% in PWID. Sexual behaviors linked to blood exposure and use of drugs may contribute to the high prevalence among MSM and HIV positive MSM. Chemsex, referring to voluntary intake of psychoactive and non-psychoactive drugs to facilitate and/or enhance sexual intercourse mostly among MSM, has been shown to be associated with higher risk of HIV and HCV transmission and contribute to increased risk among MSM (75).

High prevalence rates of HBV and HCV were shown among PWID in this analysis, corresponding to rates found in the EU/EEA ranging from 0.5 to 6.1% (HBsAg) and 13.8 to 84.3% (anti-HCV), respectively (61).

This coincides with the pattern of IDU being the main driver of HCV transmission in Europe accounting for more than 40% of new reported infections where the transmission route is known (76). A recent modeling study found that if the increased risk of HCV transmission among PWID was removed, an estimated 43% (95% CrI 25–67) of incident HCV infections globally would be prevented from 2018 to 2030 (77), and the population attributable fraction was higher in high-income countries. Focusing on prevention, testing, and treatment of PWID is important in targeted settings as part of harm reduction services.

In total, 14.3% of the prison population throughout Germany were anti-HCV positive (48). In the EU/EEA some of the highest rates of anti-HCV are detected among prison populations (4.3–86.3%). Further, 21.9% of the included prison populations were PWID demonstrating the intertwined relationship between at risk-groups. However, recent data are missing.

This paper aimed to describe the prevalence among GP and at-risk populations in Germany. This is however a simplistic approach given that populations at higher risk of VH may be exposed to several risk factors contributing to their vulnerability such as migration from a high prevalence country and sex work or prisoners who are sentenced due to IDU combined with potentially lack of access to health care services. Large-scale studies that focus on at-risk populations may determine differences in the prevalence of VH and identify frequent intersections between different at-risk groups in order to identify sub-populations in particular need of intensified testing and treatment efforts.

Some at-risk populations were missing in the identified literature including sex workers, persons with frequently changing sex partners, recipients of blood transfusions, and persons with tattoos and piercings. This indicates a need for more research to generate valid estimates of the prevalence in these groups to know the true burden of VH in Germany.

### Methodology—Strengths and Limitations

The broad search string used in this overall scoping review ensured that all relevant outcomes were included and reviewed. By running the search string also in CC Med Base Bielefeld, it was ensured that evidence published only in German was included. Almost half of the identified publications in the “prevalence” category were published in German (24 of 51 publications), which highlights the need to search for publications in both German and English to gain insights into ongoing research and results from Germany.

The quality of the evidence was overall good with risk of bias being low in the majority of the included publications. We used the tool developed by Hoy et al. (8) specifically developed with the purpose of assessing risk of bias in prevalence studies with the focus on looking at the attempt made by the studies in minimizing the risk of bias. The majority of the studies were not population-based prevalence surveys aiming to estimate the national prevalence of HBV, HCV, or HDV, but rather studies with non-probability based sampling methods and small sample sizes. Therefore, they failed to address some of the critical items necessary to reduce bias as set forth in the risk assessment tool by Hoy et al. Although the results were not analyzed based on the risk of bias, this was an important step in order to allow critical interpretation of data and be aware of their strengths and limitations.

Our scoping review has limitations. There is a risk of publication bias and delays in the available and published data. Attempts were made to compensate this by including non-published articles from the RKI Epidemiological Bulletin (EpiBull) and relevant regional journals. Moreover, a manual search was performed of reference lists in the included publications of the overall scoping review on VH epidemiology (6), and 14 references were identified but none were on prevalence. Further efforts, such as conducting a search for gray literature through other sources would potentially increase the number of relevant non-published literature.

This analysis was part of a large comprehensive review covering all aspects of viral hepatitis B, D, and D epidemiology in Germany and presents data on VH prevalence until 2017 (6). With this comprehensive review, information on the baseline situation which is necessary for better monitoring of VH elimination in Germany was collected. The time period before 2017 is of special interest as it serves as baseline to identify where the evidence gaps are and where the prevalence data are missing. An update of the overall review, including prevalence data, is planned to be conducted within the next few years, where the current review will serve as baseline.

Comparisons between the publications in this analysis are challenging because of their heterogeneity. The publications have made use of different study design, population, age-groups, and marker etc. which hinders the drawing of conclusions on patterns and temporal trends of prevalence. Similarly, geographical trends were not possible to analyse due to too few publications with same methodology from the same regional areas in Germany.

Publications with self-reported data and data where the diagnostic marker was not specified were included in this review. However, it is important to emphasize that these cannot be compared to studies based on laboratory data and data with specific diagnostic markers. Therefore, they are mentioned in the text and Table 2 as our aim with the review was also to outlay where there is evidence and where there is not, but excluded from the figures as direct comparisons are not possible.

The majority of the studies included were large cross-sectional screening studies in which patients attending general practices or emergency rooms were offered screening for VH. There is a gap in evidence from longitudinal studies, which could contribute to an understanding of how the VH epidemic is evolving and would allow calculation of incidence and the effects of prevention and control measures on reaching the VH elimination targets. Differences identified in this review are more likely the results of heterogeneous methodology rather than reflections of changes in the VH epidemic. Nonetheless, blood donors represent a group for which standardized data are collected nationwide and over time. The six studies included in this review covered the period from 2005 to 2010, and throughout this 5 years' time period the HBV and HCV prevalence was low, and slightly lower in the later years [2005: 0.14% (HBV), 0.08% (HCV), 2010: 0.12% (HBV), 0.07% (HCV)].

During the time period in which the evidence identified in this review was published, the assays used to test for VH have changed. This may have contributed to a difference in prevalence found in the different studies. In particular for anti-HBc where patterns need to be carefully evaluated due to the risk of differences in sensitivity with the more recent tests having a higher sensitivity than the older tests.

It is also important to underline that some HBsAg positive may be inactive chronic carriers and thereby not sick, eligible for treatment or at risk for developing sequelae. When screening people with migrant background, in particular, many inactive HBsAg carriers with low viremia are identified. However, although not eligible for clinical treatment inactive HBsAg carriers can still transmit the virus to other persons. In this review, of the 39 publications that reported on HBV prevalence, 11 reported on either HBV DNA or HBeAg among those testing HBsAg positive. Further, screening for anti-HBc is important, as while it detects past infection, HBV can reactivate in people who are immunocompromised (e.g., PLWH).

Of the 33 publications covering HCV prevalence, only 13 tested for HCV RNA in addition to anti-HCV, which is important to demonstrate chronic HCV and replication. And importantly, our results include articles published until 2017, which means that the potential impact of the highly effective direct-acting antiviral (DAAs) treatment options on the HCV epidemic are not sufficiently covered in this review.

## Conclusion

Globally, the elimination of VH is still gaining momentum. The progress of the interventions needed to reach the WHO elimination goals are being monitored (78) and the continuous need to collect strategic information to target the response is key. This review contributes to the understanding of the existing knowledge about the VH epidemic in Germany.

A comprehensive evidence-based overview of the available evidence on VH prevalence in Germany was provided. While there is overall good evidence, this is largely on HBV and HCV prevalence in the general and clinical populations. Gaps in knowledge exist for HDV and at-risk populations, and longitudinal studies are needed to uncover trends in the epidemic. Although Germany is considered a low prevalence country, high and very high rates are found among at-risk populations, in particular among PWID. Further research is needed on these groups and representative samples at the national level to gain much needed insights into the large-scale patterns of VH and the progress toward reaching the WHO elimination goals by 2030 in Germany.

## Author Contributions

RZ and VB conceptualized this study. SD supervised the study. GS, NS, and SL carried out the study. GS, NS, and RZ developed the research questions and drafted the a priori protocol. GS, NS, SL, SB, RT, RZ, and SD extracted the eligible data. IS and SD performed the analyses. IS, GS, NS, MC, HW, YS, VB, RZ, and SD interpreted the results and contributed to the discussion. IS drafted the manuscript. All authors critically revised the manuscript and approved the final version.

## Conflict of Interest

The authors declare that the research was conducted in the absence of any commercial or financial relationships that could be construed as a potential conflict of interest.

## Supplementary Material

Supplementary S1 Filename: S1_Text_search strategy.docx. Title of data: Search strategy. Description of data: The search strategy used for the study to identify the included publications.

Supplementary S2 In- and exclusion criteria. Filename: S2_Figure_In- and exclusion criteria.tiff. Title of data: In- and exclusion criteria. Description of data: Table listing the criteria for selection of the publications retrieved through the search.

## Abbreviations

DAAs: Direct-acting antiviral treatment
BASE: Bielefeld Academic Search Engine
CC Med: Current Contents Medizin
DEGS 1: National population-based survey among adults in Germany
GP: General population
EMBASE: Excerpta Medica Database
Europe PMC: Europe PubMed Central
HCWs: Health care workers
IDU: Injecting drug use
IMIRA: Improving Health Monitoring in Migrant Populations
MEDLINE: Medical Literature Analysis and Retrieval System Online
MSM: Men who have sex with men
PICO: Participants, Interventions, Comparator, Outcome
PWID: People who inject drugs
PLWH: People living with HIV
PLWVH: populations with non-VH related underlying disease and people with VH in hepatological care
PRISMA-ScR: Preferred reporting items for systematic reviews and meta-analysis extension for scoping reviews
PROSPERO: International prospective register of systematic reviews
RKI: Robert Koch Institute
VH: Viral hepatitis.
