# Supplementary material for: Prevalence of Hepatitis B, C, and D in Germany: Results From a Scoping Review
Source: Front Public Health. 2020 Aug 28;8:424. doi: 10.3389/fpubh.2020.00424 (PMC7493659; doi:10.3389/fpubh.2020.00424)
Supplement: Supplementary S1 — Filename: S1_Text_search strategy.docx. Title of data: Search strategy. Description of data: The search strategy used for the study to identify the included publications. [file Table_1.pdf]

S1_Text: Search strategy

1. **Search in Medline, EMBASE, Europe PMC, Scopus (full-text search; date of last search: 9 March 2017, search restricted to 1 January 2005 – 9 March 2017; filters: English and German language):**

#1 hepatitis b
#2 HBV

#3 hepatitis c
#4 HCV

#5 hepatitis d
#6 HDV

#7 viral hepatitis

#8 European Union
#9 EU
#10 EEA
#11 europ*
#12 german*
#13 deutsch*
#14 Berlin
#15 Hamburg
#16 Munich
#17 Cologne
#18 Frankfurt
#19 Stuttgart
#20 Dusseldorf
#21 Dresden
#22 Dortmund
#23 epidemiol*
#24 seroepidem*
#25 screen*
#26 prevalence*
#27 seroprevalence*
#28 incidence*
#29 "burden of disease"
#30 "disease burden"
#31 burden
#32 morbid*
#33 DALY*
#34 mortality*
#35 letal*
#36 risk factor*
#37 transmission
#38 infection risk*
#39 viraemic rate*
#40 infection rate*
#41 therapy rate*
#42 healing rate*
#43 reinfection*
#44 vaccination rate*
#45 vaccination coverage

#46 modelling
#46 epidemiology
#47 #1 OR #2 #7
#48 #3 OR #4 OR #7
#49 #5 OR #6 OR #7

#50 #8 OR #9 OR #10 OR #11 OR #12 OR #13 OR #14 OR #15 OR #16 OR #17 OR #18 OR #19 OR #20 OR #21 OR #22

#51 #23 OR #24 OR #25 OR #26 OR #27 OR #28 OR #29 OR #30 OR #31 OR #32 OR #33 OR #34 OR #35 OR #36 OR #37 OR #38 OR #39 OR #40 OR #41 OR #42 OR #43 OR #44 OR #45 OR #46

#52 #23 OR #24 OR #25 OR #26 OR #27 OR #28 OR #29 OR #30 OR #31 OR #32 OR #33 OR #34 OR #35 OR #36 OR #37 OR #38 OR #39 OR #40 OR #41 OR #42 OR #43 OR #46

#53 #47 AND #50 AND #51
#54 #48 AND #50 AND #52
#55 #49 AND #50 AND #51

2. **Search in CC Med (date of last search: 9 March 2017, search restricted to 2005 – 2017; filters: German language):**

#1 hepatitis b
#2 HBV
#3 hepatitis c
#4 HCV
#5 hepatitis d
#6 HDV
#7 virale hepatitis

#8 epidemiolog*
#9 seroepidemiolog*
#10 prävalenz
#11 seroprävalenz
#12 inzidenz
#13 mortalität
#14 morbidität
#14 krankheitslast
#15 folgeerkrankung*
#16 letal
#17 übertragung
#18 diagnoserate
#19 therapierate
#20 infektion
#21 impf*

#22 #1 OR #2 OR #7
#23 #3 OR #4 OR #7
#24 #5 OR #6 OR #7

#25 #8 OR #9 OR #10 OR #11 OR #12 OR #13 OR #14 OR #15 OR #16 OR #17 OR #18 OR #19 OR #20 OR #21
#26 #8 OR #9 OR #10 OR #11 OR #12 OR #13 OR #14 OR #15 OR #16 OR #17 OR #18 OR #19 OR #20

#27 #22 AND #25
#28 #23 AND #26
#29 #24 AND #25

3. ***Search in Base Bielefeld (date of last search: 9 March 2017, search restricted to 2005 – 2017; filters: German language):***

#1 Hepatitis B
#2 HBV
#3 Hepatitis C
#4 HCV
#5 Hepatitis D
#6 HDV
#7 virale Hepatitis
#8 deutsch*
#9 germ*

#10 #1 OR #2 OR #7
#11 #3 OR #4 OR #7
#12 #5 OR #6 OR #7

#13 #8 AND #9

#14 #10 AND #13
#15 #11 AND #13
#16 #12 AND #13
