# Supplementary material for: Prevalence of Hepatitis B, C, and D in Germany: Results From a Scoping Review
Source: Front Public Health. 2020 Aug 28;8:424. doi: 10.3389/fpubh.2020.00424 (PMC7493659; doi:10.3389/fpubh.2020.00424)
Supplement: Supplementary S2 — In- and exclusion criteria. Filename: S2_Figure_In- and exclusion criteria.tiff. Title of data: In- and exclusion criteria. Description of data: Table listing the criteria for selection of the publications retrieved through the search. [file Table_2.pdf]

S2_Figure: In- and exclusion criteria

Inclusion criteria	Exclusion criteria
**1. Type of publication:**<br>**1.1.1. publications:** only if full text available<br>Peer-reviewed article, non-Peer-reviewed article, report, dissertation<br>**1.1.2. non-published data**<br>published and non-published data without further explanation only after consulting the data provider (e.g. secondary data, registry data)<br><br>**2. Type of work:** only original work with a clear origin of the analysed data<br><br>**3. Type of data:** original data (observational studies, e.g. cross-sectional studies, cohort studies, cases of case-control studies), reviews with a systematic approach/evidence based guidelines with a underlying systematic review, secondary/registry data<br><br>**4. Quality standards:** report of n study population, age groups and sex study population, place of data collection<br><br>**5. Time frame**<br>data collection not finished before 1/1/2005 published after 1/1/2005<br>**1. Content:**<br><br>1.1. Relation to:<br>- Hepatitis B, C und D<br>- Germany<br>- Humans<br><br>1.2. Epidemiological relevance:<br>distribution, prevlanece, incidence, mortality, morbidity related to<br><br>**1.2.1.** Hepatitis B, C und D infection<br>**1.2.2.** sequelae (only acute liver failure, hepatocellular carcinoma, liver cirrhosis)<br>**1.2.3.** co-Infection: only HIV and HBV/HCV/HD<br>**1.2.4.** distribution of subtypes<br>**1.2.5.** transmission routes<br>**1.2.6.** quality of life<br><br>1.3. Data related to:<br>**1.3.1. diagnosis rate** (proportion of diagnosed infections, screening)<br>**1.3.2. therapy rate** (indicated, started, finished, interrupted)<br>**1.3.3. vaccination coverage**<br>**1.3.4. transplantation rate** directly related to Hepatitis B, C, D	**1. Type of publication:**<br>**1.1. publications**:<br>**No** full text existing or available for study team<br>**1.2. Non-published data**<br>**No** consultation of the data provider possible<br>**1.3. Data already used in other publication:**<br>Publication without further information regarding the study questions<br><br>**2. Type of work:** *no* original work<br>e.g. expert opinions, narrative reviews, editorials, comments<br><br>**3. Type of data:** e.g. method papers (containing no data), case series, RCT<br><br>**4. Quality standards:** No report of: n study population, age groups and sex study population, place of data collection<br><br>**5. Time frame:** data collection finished before 1/1/2005 published before 1/1/2005<br><br>**6. Publications reporting identical outcomes of the same study and no new and relevant aspects according to the research questions (newer publication excluded)**<br><br>**1. Content:**<br><br>**1.1.** No relation to :<br>**1.1.1.** Hepatitis B, C und D<br>**1.1.2.** Germany<br>**1.1.3.** Humans<br><br>**1.2.** No relevance for study question:<br>**1.2.1.** other topics than in study questions<br>**1.2.2.** pathogen: virology, clinical symptoms<br>**1.2.3.** other sequelae than acute liver failure, hepatocellular carcinoma, liver corrhosis<br>**1.2.4.** other co-infections than HIV and HBV/HCV/HDV<br><br>**1.3.** Data related to:<br>**1.3.1. diagnosis**: diagnostics, lab methods<br>**1.3.2. therapy**: single drugs, therapy scheme, therapy in immunocompromised patients, side effects<br>**1.3.3. vaccination**: vaccines, vaccination schedules, vaccination of immunocompromised, side effects<br>**1.3.4. liver transplantation** (LTX): risks, process, complications, drug therapy, outcome, re-transplantation, LTX under specific circumstances<br><br>**1.4.** Data related to:<br>**1.4.1.** prevention<br>**1.4.2.** level of care<br>**1.4.3.** costs<br>**1.4.4.** politics
